# Roles of MicroRNA across Prenatal and Postnatal Periods

**DOI:** 10.3390/ijms17121994

**Published:** 2016-11-28

**Authors:** Ilaria Floris, Jamie D. Kraft, Illimar Altosaar

**Affiliations:** Biochemistry, Microbiology & Immunology Department, Faculty of Medicine, University of Ottawa, 451 Smyth Road, Ottawa, ON K1H8M5, Canada; ilaria.floris84@gmail.com (I.F.); jkraf053@uottawa.ca (J.D.K.)

**Keywords:** maternal-offspring crosstalk, microRNA, noninvasive biomarkers, breast milk

## Abstract

Communication between mother and offspring in mammals starts at implantation via the maternal–placental–fetal axis, and continues postpartum via milk targeted to the intestinal mucosa. MicroRNAs (miRNAs), short, noncoding single-stranded RNAs, of about 22 nucleotides in length, are actively involved in many developmental and physiological processes. Here we highlight the role of miRNA in the dynamic signaling that guides infant development, starting from implantation of conceptus and persisting through the prenatal and postnatal periods. miRNAs in body fluids, particularly in amniotic fluid, umbilical cord blood, and breast milk may offer new opportunities to investigate physiological and/or pathological molecular mechanisms that portend to open novel research avenues for the identification of noninvasive biomarkers.

## 1. Background

In developing mammals, the vulnerability and immature immune status of the fetus and newborn are compensated by maternal factors that are transferred via the placenta or amniotic fluid, during intrauterine life, and by milk after birth [1].

During development, mother-fetal cross-talk begins at implantation, via the maternal–placental–fetal axis [1]. The biological dialogue initiates between the maternal reproductive tissue and the conceptus [2]. The placenta plays a fundamental and regulatory role by producing a wide variety of hormones, growth factors, and cytokines [3], as well as other biologically active molecules, including miRNA [4,5]. These biomolecules act both locally and systemically, exchanging information and regulating the physiological and metabolic processes required during intrauterine fetal life [3,4,5]. The intrauterine environment strongly influences growth and fetal development, in addition to the health status of both the mother and child long after birth [5,6,7,8].

During fetal development, the gastrointestinal (GI) tract is characterized by higher permeability which may allow amniotic fluid to be absorbed by the fetus. Both amniotic fluid and breast milk have nutritive, protective, and regulatory roles [9]. Strikingly, amniotic fluid administered postpartum in the preterm piglet model is comparable to colostrum in its ability to modulate intestinal inflammation, to increase body weight, to alter bacterial colonization, and to induce differential expression of mRNA coding for genes involved in gut inflammatory responses [10].

After birth, the mother’s milk continues to provide oral biochemical signaling previously provided to the fetus via amniotic fluid [1]. Milk undoubtedly offers the nutritional requirements for growth and infant development, but it is much more than food, providing protection and long-term programming agents for the infant [11]. The term “lactocrine” has been proposed to indicate the comprehensive maternal signals sent to the offspring via milk [12].

The biochemical factors strongly influencing offspring development in both pre- and postnatal periods are interpreted as an ancient adaptation of mammals, starting with the origin of lactation [13,14], and enhanced with the evolution of the placenta. Particularly, human infants are born much earlier than nonhuman primates and are in need of additional support. For example, comparative proteome studies in humans and rhesus macaques revealed that human milk is richer in proteins involved in the development of the GI tract, the immune system, and the brain [15]. Indeed, lactation strategies and milk composition vary among species and individuals, reflecting the biological process of natural selection [16,17]. In this review, after summarizing the current knowledge of miRNA expression and potential participation in the regulation of various cell mechanisms, we will present recent findings suggesting that miRNAs are involved in maternal-infant crosstalk during both pregnancy and suckling periods.

## 2. Micro-Intro to the MicroRNA World

miRNAs are short, noncoding, single-stranded RNA of about 22 nucleotides in length. They are transcribed in the nucleus from miRNA genes as longer hairpin precursors known as primary miRNA, which are processed by the endoribonuclease Drosha to form precursor miRNA. These are exported by Exportin-5 into the cytoplasm, where they are further processed by the endoribonuclease Dicer to form mature miRNA duplexes [18,19]. The duplex consists of a guide strand and a messenger strand of which the latter ultimately is degraded and the former becomes a mature miRNA. The mature miRNA becomes part of the RNA-induced silencing complex (RISC) after being loaded onto Argonaute. Through partial complementation, miRNA becomes a guide for the RISC to target the 3′ untranslated region of messenger RNA (mRNA) [18]. Their main function is to repress target mRNA via translation inhibition and/or transcript degradation, and to silence gene expression [19]. A single miRNA can bind and target multiple mRNAs and in turn, one mRNA can be regulated by multiple miRNAs [20,21]. Transcription, translation, and protein degradation are prominent examples of how gene activity is controlled by miRNAs, considered to be “meta-regulators” [22]. Importantly, miRNAs can target epigenetic regulators, which play a role in fetal metabolic programming [23,24], mediating long-term effects on target cells [24,25], and on developing organs and tissues. Epigenetic factors and miRNAs have been shown to be reciprocally regulated [24,25]. Thus, miRNAs are recognized as key regulators of diverse biological and developmental processes in eukaryotes (cell proliferation and differentiation, maintenance of tissue identity, apoptosis, immune system development, and responses) and are associated with pathologies, including different types of cancer [26], vascular diseases [27,28], and diabetes [24,29].

Nomenclature and other important information about precursor hairpin sequences, and experimentally identified mature miRNA sequences, are available online in a database called miRBase (http://www.mirbase.org/). In October 2016, miRBase had a total of 28,645 entries of hairpin precursor miRNAs profiled in a vast range of plant and animal species. For *Homo sapiens* we counted 1881 precursors and 2588 mature miRNAs. In addition, miRBase provides links to databases for validated miRNA targets (TarBase) and miRNA target prediction (DIANA-microT, microRNA.org, miRDB, RNA22, TargetMiner, PicTar-vertebrates) for in silico studies. Other in silico tools are progressively becoming available for functional miRNA analysis. For example, MAGIA83 (miRNA And Genes Integrated Analysis) (http://gencomp.bio.unipd.it/magia/start/) integrates expression profiles of miRNA and mRNA to investigate more deeply into biological networks and pathways. Online databases, such as miR2disease86 (http://www.mir2disease.org/), can display miRNA expression profiles in human disease scenarios. Ingenuity Pathway Analysis (IPA) is a commonly used tool to identify mRNAs as targets for miRNAs in silico.

miRNAs are expressed not only in tissues and cells, but also in all body fluids tested: milk, plasma, urine, saliva, tears, seminal fluid, amniotic fluid [4], and umbilical cord blood [30]. RNA molecules were once considered to be very unstable due to the presence of ribonucleases (RNases), which are abundant and ubiquitous to neutralize viral and bacterial nucleic acids. However, RNA molecules are now known to circulate in a “safe and stable” manner in body fluids [4]. RNases are implicated in various functions of innate and acquired immunity, in stimulating host defense, and in sustaining mucosal barriers [31]. In early 1988, Steven Benner proposed a hypothesis regarding “extracellular communicator RNA”. He suggested that RNA molecules might be messengers involved in cell–cell communication, the balance between RNase/RNase inhibitors. He also proposed that RNA molecules were related to diseases, including cancer and angiogenesis [32]. During the following 20 years, researchers around the world working in different fields, continued to investigate RNA control of gene expression [33] and its role in cell-to-cell signaling [34]. Today we are aware that extracellular noncoding RNA, such as miRNA and long non-coding RNA (lncRNA) [35,36], are involved in cellular communication, may be involved in childhood development, and are protected from RNases through association with RNA-binding proteins and/or by their encapsulation inside extracellular vesicles [36,37,38].

During the last decade, extracellular vesicles (EV) have been studied thoroughly regarding their biogenesis, content, and biological function. They can mediate the transfer of proteins, lipids, and nucleic acids, including miRNAs and lncRNAs [36], and have been recognized as potent vehicles of intercellular communication, both in prokaryotes and eukaryotes [39]. EV have biological functions linked to sperm maturation and motility, follicular growth, oocyte meiosis, steroidogenesis, and the prevention of polyspermy after fertilization. In the uterus, EV mediate the crosstalk between embryo and endometrium during implantation [40] and are crucial regulators throughout pregnancy [41,42].

## 3. New Kids on the Block: miRNAs Really Do Matter

**Figure 1:**
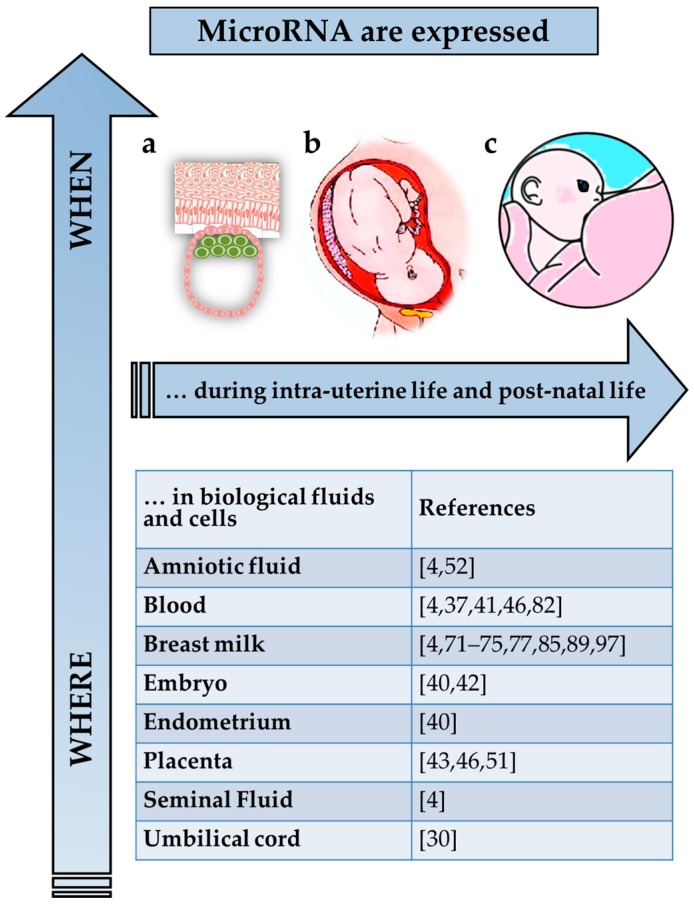
MicroRNA in the maternal-offspring biologic interchange. MicroRNA are expressed in cells and body fluids continuously during intra-uterine (**a**,**b**) and post-natal life. After birth, the dynamic, bidirectional interchange of bioactive components, including specific miRNA, continues and can occur through breastfeeding. (**c**) miRNAs expressed constitutively and those which are expressed in a tissue-specific manner in response to environmental stimuli remains to be determined.

During the dynamics of pregnancy, miRNAs appear to play important regulatory roles in maternal-fetal crosstalk [5]. miRNAs are involved in endometrial receptivity, implantation, placental function, and labor in eutherian organisms [43,44]. Once fertilization has taken place it may be possible that seminal fluid or sperm cell miRNAs affect gene expression in the zygote, with downstream effects on successful implantation. The abundance and identity of miRNA that may be present in a single spermatocyte remains unknown [2]. miRNAs contribute to establishing immune tolerance at conception [45]. Altered immune-regulatory miRNAs during preconception and the first trimester are indicative of later pregnancy outcomes in humans (*n* = 48) [45]. During placental development, miRNAs can bind to target genes responsible for cellular invasion, proliferation, apoptosis, and angiogenesis [44]. Placenta and maternal plasma are characterized by specific miRNA patterns [46]. Many of these miRNA genes form clusters, likely controlled by the same promoters to work in a synergistic manner, which are mainly expressed during pregnancy [47]. Their rapid decrease after parturition suggests a placental-fetal origin and an expression pattern that is specifically induced during pregnancy [48]. The regulation of miRNA expression remains to be determined as some miRNAs are expressed constitutively and others are expressed in a tissue-specific manner in response to environmental stimuli. Notably, miRNAs contribute to normal term and pre-term labor. For example, in a study with 17 patients (8 term patients, 9 spontaneous term labour patients), expression of miR-223 and miR-34 was induced in the cervix during term parturition [48]. In a murine model, expression of the miR-200 family acts to control uterine quiescence and contractility via regulation of the progesterone receptor as well as the transcription factors ZEB1 and ZEB2, which was further validated using myometrial biopsies from pregnant women subject to caesarean section, both during and prior to active labour [49]. Interestingly, the fetal genome, including loci for miRNA, can generate molecular signals that are sent through the placenta and amniotic fluid into maternal circulation [50]. In addition to endogenous miRNA, exogenous non-coding RNA consumed by the mother can cross the placenta and gain access to the fetus, regulating fetal development [51]. miRNA profiles in amniotic fluid reveal their involvement in pathways (such as axon guidance, focal adhesion, and mitogen-activated protein kinase signaling pathways) related to the normal development of the nervous system and other organs (Figure 1) [52]. Even though the effect of labour on miRNA profiles has been reported in plasma, miRNA profiles have yet to be identified in milk or amniotic fluid based on vaginal or caesarean-section modes of delivery [53]. The mode of delivery has been shown to affect the bacterial presence and abundance in human milk [54]. This suggests potential for differing miRNA expression in milk depending on the mode of delivery, however, this intriguing aspect remains to be explored [55]. 

Early enteral feeds appear to have long-term health effects regarding epigenetic mechanisms. Interestingly, in mice, a low protein diet from weaning resulted in an altered miRNA profile (miR-98, miR-199, miR-21, let-7, and miR-210), most of which are involved with cellular proliferation [56]. Recent studies have revealed miRNA involvement in chromatin remodeling. Gene expression may be regulated by intertwined mechanisms of miRNAs, DNA methylation, and histone modification [56]. Previously, research focused on the changes in DNA methylation based on diet, however, there is now an increase in research emphasizing the role of nutrition in the variation of miRNAs [57]. In a sheep study, undernourishment at the time of conception led to altered miRNA expression in the skeletal muscle of offspring which was suggested to be linked to insulin resistance development [58]. Additionally, a sheep model of maternal obesity also lead to differentiation of fetal miRNA expression in muscles thought to intensify intramuscular adipogenesis [59]. These variations in miRNA profiles could be due to the signaling from the mother in utero via amniotic fluid. However, more studies need to be conducted to demonstrate the direct effects of maternal health on the miRNA profiles of offspring.

## 4. miRNA-Associated Pregnancy Complications

While assessing miRNA roles in normal pregnancy, one cannot overlook the miRNAs associated with pregnancy-related pathologies including gestational diabetes (GD) [24], implantation failure, pre-eclampsia, preterm labor, and intrauterine growth restriction [44]. GD, a glucose intolerance first recognized during pregnancy, is an independent risk factor of disease in both offspring and mother [60,61]. The hyperglycemia occurring during pregnancy induces long-term phenotypic alterations in fetal endothelial cells, changes in miR-101 expression levels, as well as in those of Enhancer of Zester Homolog 2 (EZH2), its target [24]. EZH2 is the only protein of the Polycomb Repressor Complex 2 (PRC2), known to date, to have catalytic activity [62]. PRC2 works in concert with chromatin regulators and other proteins to initiate and maintain the methylation of histone H3 on lysine 27 (H3K27), an epigenetic mark which mediates long-term gene silencing [62]. EZH2 is both a target of miR-101 and a transcriptional repressor of miR-101 via binding with the regulatory sequence(s) in the ‘miR-101 locus’ [24]. miR-101 and EZH2 are reciprocally controlled through a regulatory feedback loop. The disturbance of this homeostatic loop is associated with cell phenotypic alterations, observed after five to six passages in culture under normal glucose conditions. These alterations include lower proliferation, impaired migration and angiogenic capacity, as well as increased apoptosis [24]. Alterations of noncoding RNA, including miRNA, are implicated in persistent and long lasting epigenetic changes, that can be transferred during cell division [63], and that are associated with cell dysfunction and disease [24,63]. In diabetic conditions, the prolonged exposure to hyperglycemia generates a long-lasting impression on vascular cells and the progression of vascular complications [64], leading to the ‘glycemic memory’ theory that explains how chronic hyperglycemic conditions lead to persistent outcomes, through epigenetic changes [65] and miRNA alterations [64].

Seven miRNAs in peripheral blood (miR-1, miR-133b, miR-199a-5p, miR-1267, miR-1229, miR-223, and miR-148a-3p) are related to the predisposition for pregnancy complications such as miscarriage and early- and late-onset pre-eclampsia [45]. They have a predictive value for adverse pregnancy outcomes and therefore have a role as potential biomarkers. In addition, they are associated with low regulatory T cell numbers and low TNF/IL-10 levels, that implies an altered immunological mechanism [39]. These observations are in line with the emerging viewpoint regarding the conundrum of preterm birth, still poorly understood. Labour onset is thought to depend on the balance between maternal cells, myometrial cells, and immune cells circulating in the tissues of the mother and fetus. As there appear to be multifactorial causes for preterm birth, one systematic approach to comprehend the phenomenon and decipher its intrinsic signaling is to focus on pro-inflammatory signals and immunological pathways [66], and also explore the role(s) of related miRNAs to better understand the molecular mechanisms involved in uterine exposure to endo-/exo-genous factors [67].

## 5. Breast Milk miRNA: Possible Key Regulators and Noninvasive Biomarkers

The neonatal and infant period is substantial for growth and development, especially in humans. Human milk has the indisputable role of protecting the infant against infection and confers both short and long-term benefits, reducing morbidity and mortality while contributing to cognitive attributes [11,68,69,70]. Breast milk contains the highest concentration of total RNA of the 12 body fluids tested, more than 80 times the concentration found in amniotic fluid [4]. More than 1400 mature miRNAs are expressed in human milk [71]. These miRNAs are thought to originate from mammary gland epithelial cells [72]. Their resistance to degradation stems from their assembly with proteins (Ago2 and other RNA-binding proteins) in skim milk [72,73], packaged inside milk cells [74] and inside the heterogeneous population of milk vesicles, which include milk fat globules [75] and exosomes [76,77]. Human milk somatic cells or exosomes which carry miRNA [74] are thought to survive in the acidic environment of the stomach, to be transferred into the circulation and may then be integrated in various tissues [78]. Using a model GI tract, bovine milk exosomes have been shown to withstand the harsh conditions of the gut [79]. There are a number of known and unknown miRNAs conserved in human milk cells and lipids that are differentially expressed during lactation [80]. Exogenous food-derived miRNAs appear to be stable in the oral cavity and in the GI tract and are transferred to the blood of adults, thought to influence gene expression in different tissues [81,82,83]. Recent evidence noted that bovine milk exosomes are taken up by human intestinal cells and vascular endothelial cells via endocytosis [84]. However, more studies are required to demonstrate that milk miRNAs, once ingested, can cross the GI tract of the infant [83,85]. Recent studies have shown the resistance of miRNA to degradation and that uptake of bovine milk exosomes by intestinal cells in vitro is mediated by temperature dependent-endocytosis [79,86]. A more recent study has provided evidence that porcine milk exosomal miRNAs modify gene expression and promote proliferation in intestinal cells in vitro and in vivo [87]. Importantly, miRNAs are thought to either have a functional role or a nutritional role. The functional hypothesis suggests that milk miRNAs are absorbed by the suckling baby to imply specific actions: for example, to modulate and shape the immune system [85,88] by regulating T-cells, inducing B-cell differentiation [71,77], and preventing the development of allergies [89]. The nutritional hypothesis suggests miRNAs simply provide nutrition and refutes the transfer and the absorption of milk miRNA into the systemic circulation of offspring [85].

The fate and the role of miRNAs and other non-coding RNA components in breast milk still remain unknown. Interestingly, breast milk is rich with immune-related miRNAs [71,77,88] and miRNAs are implicated in multiple physiological processes [75], including cellular differentiation and proliferation, tissue identity, metabolism, and developmental programming [72]. Some miRNAs present in milk are involved in nervous system pathways and may mediate brain development. For example, miR-118.2 [75] targets the Teneurin Transmembrane Protein 2, a protein abundant in the central nervous system and important in neural function [90]. Some other miRNAs in human milk are tissue-specific, such as miR-142-5p [73], which is hematopoiesis-specific [91]. These findings suggest that milk miRNA may target specific organs and tissues of the feeding infant. In addition, there are milk miRNAs that are involved in metabolic pathways. For instance, miR-21 [73,75,77], appears to play an important role in promoting postnatal growth via mTORC1 signaling [92], while components of the let-7 family, also abundant in milk [73,75], are involved in glucose metabolism and can regulate glucose tolerance and insulin-sensitivity [93]. Furthermore, miR-33, miR-122, miR-370, miR-378-3p, and miR-125a-5p, abundant in the human milk lipid fraction [75] and milk exosomes [77], are potential regulators of lipid metabolic pathways in the developing infant and/or are possible factors involved in mammary gland function during lactation [94].

Where miRNAs are found to target epigenetic regulators the effect on the recipient target can be persistent and durable [24,63]. miR-148-3p, one of the most highly expressed miRNAs in human milk [75], targets the DNA methyltransferase 3b, a key component of the epigenetic machinery during development [95] and an essential DNA methyltransferase in the intestinal epithelium [96]. Through milk, the infant may benefit from these bioactive molecules, which may act as key regulators to set up important processes during the maturation of organs, such as the GI tract. The epithelium of the GI system is continuously renewed and dynamically regulated by epigenetic modifications and transcription factors, which influence intestinal cells throughout the crypt-villus axis [97]. Histone methylation and acetylation are crucial in controlling proliferation and differentiation of intestinal cells of the crypts [97]. DNA methylation sites are key regulators of postnatal epigenetic changes in intestinal stem cells [98]. Promisingly, miRNAs in milk that target epigenetic regulators involved in histone modification and DNA methylation are possible candidates for the process of gut maturation.

Daily fluctuations of mRNA [99] and miRNA [73] in human milk suggest that other physiological roles can be explored. Circadian variations of milk components, including miRNA, may help to establish and/or fine-tune gastrointestinal circadian rhythmicity of the breast fed infant, mediating what we now refer to as lactocrine circadian signals [73].

During the course of the lactation period the expression levels of miRNAs such as miR-25, miR-155, miR-182, miR-191, miR-221, and miR-223, change in the mammary glands of cows [100], pigs [101], rats [102], and humans [71]. The expression of cellular and extracellular miRNA in bovine mammary epithelial cells changes under the influence of lactogenic hormones such as prolactin [103]. These findings suggest that miRNA is implicated in the physiological processes of milk synthesis under normal conditions. In this context, it is noteworthy that the deregulation of miRNA has been associated with disease and pathological status [21,26], supporting their use as biomarkers for evaluating the performance and health status of the mammary gland during lactation [11,99]. miRNA levels in blood are measured and successfully used to predict breast cancer susceptibility [104] and other diseases [105], for example, mastitis [106]. In a similar way, miRNA in breast milk deserves to be studied in neonatal and perinatal medicine to identify biomarkers for infant diseases [11] and to better understand the unique dyadic exchange between mothers and their babies.

## 6. Conclusions

The biological dialogue between a mother and her offspring starts from the implantation of the embryo in the uterus and continues during fetal life via the maternal–placental–fetal axis [1]. In this review, we summarize the current knowledge concerning the role(s) of miRNA in the regulation of various physiological and pathological processes involved in maternal-infant crosstalk during both pregnancy and suckling periods. The expression of miRNAs is ubiquitous; they are present in cells and body fluids of both maternal and fetal origin. Here we focus on their potential participation in offspring development throughout prenatal and post-natal life. In particular, we discuss and cite studies congruent with the functional hypothesis [85], which proposes that milk miRNAs are absorbed by the GI of the suckling baby to ensure and regulate specific physiological processes.

## Abbreviations

miRNA	microRNA
EV	Extracellular Vesicles
GD	Gestational Diabetes
EZH2	Enhancer of Zester Homolog 2
PRC2	Polycomb Repressor Complex 2
H3K27	Histone H3 on Lysine_27_
GI	Gastrointestinal
